# The Short Warwick-Edinburgh Mental Well-being Scale (SWEMWBS) - A psychometric evaluation of adolescents in Sweden during the COVID-19 pandemic

**DOI:** 10.1016/j.heliyon.2024.e27620

**Published:** 2024-03-05

**Authors:** Amir H. Pakpour, Marit Eriksson, Ida Erixon, Anders Broström, Staffan Bengtsson, Malin Jakobsson, Karina Huus

**Affiliations:** a Department of Nursing, School of Health and Welfare, Jönköping University, Jönköping, Sweden; b Futurum - the Academy for Health and Care, Region Jönköping County, Sweden; c Department of Public Health and Health Care, Region Jönköping County, Sweden; d Department of Clinical Neurophysiology, Linköping University Hospital, Linköping, Sweden; e Department of Health and Caring Sciences, Western Norway University of Applied Sciences, Bergen, Vestlandet, Norway; f CHILD-research Group, School of Health and Welfare, Jönköping University, Sweden; g Department of Social Work, School of Health and Welfare, Jönköping University, Jönköping, Sweden

**Keywords:** Mental health, Confirmatory factor analysis, Rasch analysis, Differential item functioning, Short warwick-edinburgh mental well-being scale, Measurement invariance

## Abstract

The Short Warwick-Edinburgh Mental Well-being Scale (SWEMWBS) is effective in assessing positive aspects of mental health. Despite its advantages, little is known about group differences in the interpretation of SWEMWBS items across age groups, especially during the adolescence period. Hence, this study examined the psychometric properties of the SWEMWBS through Confirmatory Factor Analysis (CFA), Rasch analysis and network analysis of Swedish adolescents during the COVID-19 pandemic. A total of 5548 participants from the south of Sweden (i.e., Jönköping County) took part in this cross-sectional study through an online platform between September 2020 and October 2020. The CFA, Rasch (including differential item functioning, DIF) analysis and network analysis were used to examine the psychometric properties and measurement invariance of the SWEMWBS. The SWEMWBS had a unidimensional structure with robust psychometric properties. The CFA demonstrated measurement invariance across gender, school year and country of birth, which was also confirmed by Rasch DIF. Furthermore, considerable associations between the items of the SWEMWBS, general health and COVID-19 impact items were observed in network analysis. The SWEMWBS showed robust psychometric properties capable of assessing positive aspects of mental health and well-being among adolescents.

## 1 Introduction

The prevalence of children and adolescents with poor mental health has increased across European regions [1]. According to a recent report from UNICEF [2], nine million European adolescents between 10 and 19 years old suffer from mental health disorders (especially anxiety and depression). Because adolescents are especially vulnerable to adverse negative consequences, insufficient attention to their mental health can significantly disrupt family, social, and school functions.

Mental well-being is a multidimensional concept and is defined as an individual's subjective sense of well-being and overall satisfaction with life, which encompasses emotional, psychological, and social well-being [3]. Theoretically, two fundamental aspects develop mental well-being: hedonic and eudemonic. The subjective experience of happiness and life satisfaction can shape the hedonic aspect. In the eudemonic aspect, an individual's psychological function, sense of purpose, and personal growth are considered [4]. To measure mental well-being, a valid and reliable scale is needed. Many different scales have been developed to measure mental well-being [5]. Each of these tools assesses a specific aspect of mental well-being. For instance, the Positive and Negative Affect Schedule focuses on the hedonic or positive affect dimension. Therefore, a tool that can address both dimensions of mental well-being has superiority over others. The Warwick-Edinburgh Mental Well-being Scale (WEMWBS) captures both aspects of mental well-being components [6] and it has demonstrated robust psychometric properties through different cultures with a variety of populations and settings, including primary care, community samples, and employee samples [7]. The WEMWBS is a 14-item self-report scale that measures both hedonic and eudaimonic aspects of mental health, including happiness, satisfaction with life, and ability to cope with stress. Therefore, the WEMWBS tries to cover both emotional and functional dimensions of mental wellbeing. Previous studies on the WEMWBS have demonstrated its validity, reliability, and ease of completion among diverse populations across various cultures. Despite past efforts to confirm the unidimensional structure of the WEMWBS [6], its factor structure remains controversial in the literature [8]. While most studies investigating the factor structural of the 14-item WEMWBS endorse the single-factor model [[9], [10], [11], [12], [13], [14], [15], [16]], some accept this structure with modifications, such as adding covariances between the error terms for some specific items [[17], [18], [19], [20], [21], [22], [23]]. This is not in line with the multidimensional theoretical framework of wellbeing. Furthermore, some studies reject the single-factor model in favor of a two- [24,25] or three-factor [26], or even a bifactor structure [8,27]. Consequently, there is not a clear factorial structure of the 14-item WEMWBS, suggesting a need for developing a short version to avoid item redundancy. The short version of the Warwick-Edinburgh Mental Well-being Scale (SWEMWBS) has been developed by Rasch analysis [28] and consists of seven of the 14 items in the original scale. The SWEMWBS has predominantly emphasized eudaimonic aspects and has been found to demonstrate preferable psychometric properties when compared to the full 14-item version [28].

The findings from over 18 previous studies indicate a consistent and stable factorial structure for the short version of WEMWBS across diverse populations. Most of these studies endorse the single-factor structure of this scale [12,14,16,17,19,20,23,[29], [30], [31], [32], [33], [34], [35], [36], [37], [38], [39]]. However, a recent study focusing on a representative Finnish adult population identified the bifactor structure as the optimal model for the SWEMWBS [26]. Consequently, the researchers concluded that, while the unidimensional structure remains applicable for assessing overall mental well-being, it should be approached with certain limitations in mind. Therefore, it would be valuable to explore whether the SWEMWBS can still be considered unidimensional.

There are two different approaches when assessing psychometrics; CTT (Classical Test Theory) and IRT (Item Response Theory) [40]. CTT is a traditional psychometric approach to measure a scale and it assumes that test scores are a combination of true scores and error scores. CTT however has several limitations, including not considering item and individual characteristics, not providing estimates of reliability or measurement invariance, and assuming a normal distribution and additivity of scores. The IRT, in turn, can be used for improving the measurement properties and construct validity of scales. The Rasch model is a specific type of IRT that can be used to evaluate the fit of the data, estimate person and item parameters, and evaluate measurement invariance across groups. Therefore, IRT can be used in conjunction with CTT to cover some of the limitations of CTT [41]. Rasch analysis is a unidimensional measurement model that is used to evaluate the properties of individual items on a scale and the relationship between the scale and the underlying latent variable [42].

Currently, the SWEMWBS has been extensively investigated in at least 12 psychometric published studies, comprising more than six languages worldwide with findings supporting its adequate psychometric characteristics [17,[35], [36], [37], [38]]. The SWEMWBS is a highly recommended psychometric test for helping experts in assessing mental well-being, and its usefulness linked to adult populations has previously been validated in relation to the Swedish context [33]. At the same time, no known study has yet examined the psychometric properties regarding uniformity and differences (measurement invariance: MI), of the Swedish version of the SWEMWBS among adolescents. Gender and age differences are crucial factors in measuring psychometric properties. Previous studies have indicated that WEMWBS has demonstrated invariance across age groups [29,35] but not consistently across gender groups [28,29]. Furthermore, immigration can have a negative effect on the mental well-being of adolescents [43,44]. For example, life dissatisfaction was more prevalent among adolescents from first- and second-generation immigrant backgrounds compared to their European native peers [44]. Therefore, adolescents with diverse immigration backgrounds may interpret the SWEMWBS differently based on their unique experiences and cultural perspectives. Assessing MI can help to ensure meaningful comparisons of the SWEMWBS across diverse subgroups such as gender or immigration background [45]. The examination of the psychometric properties of the SWEMWBS using measurement invariance and network analysis techniques can provide valuable insights into the relationships between different items of the scale and self-reported general health, as well as identify key factors that contribute to mental well-being among adolescents. Such findings could inform the development of interventions, support the use of the SWEMWBS to assess mental well-being in Swedish youths, and support strategies to promote mental well-being among adolescents in Sweden.

Hence, this study examined i) the psychometric properties of the SWEMWBS among Swedish adolescents; and ii) the measurement invariance of the SWEMWBS across gender, school year, and country of birth.

## 2 Methods

### 2.1 Participants and procedure

In this research, 8379 students in school year 9, aged 14–15, in compulsory school and in the second grade of upper secondary school, aged 17–18, in Jönköping County in the south of Sweden were invited to participate in an online cross-sectional survey study. Of these, 6454 (77%) chose to participate and 5548 (66% of the invited students) with complete data on the studied variables were included in the present study. Data was collected using a web-based questionnaire, which was administered in the schools and answered anonymously during a class supervised by the usual teacher during the period September to October 2020. Participants received an online invitation containing an URL link to the questionnaire and were asked to complete it.

The Swedish Ethical Review Authority approved the study procedures (Dnr 2020–03173). All participants needed to read the study aims and descriptions and give consent before participating in the study. In accordance with the Swedish Code of Statutes (2003:460, § 18), if a participant is between 15 and 18 years of age and can independently understand what the research entails, he or she can consent to be included without parental consent. The inclusion criteria were being aged 15 years old and more, and studying in a school in Jönköping County. Informed consent was obtained from all subjects.

The study included 5548 participants from Sweden aged between 15 and 18 years. Over 50% of the participants were boys (n = 2822). Over 83% of the participants (n = 4633) had been born in Sweden and 69% of the participants (n = 3834) reported that they or their significant others had had COVID-19 infection. About 55% (n = 3076) of the participants were studying in year 2 at upper secondary school. Missing data was less than 2.5% for all SWEMWBS items.

### 2.2 Measures

Sociodemographic data: all participants provided sociodemographic data pertaining to gender and country of birth.

*Short version of the Warwick-Edinburgh Mental Well-being Scale*: The SWEMWBS [6] is made up of seven items that assess mental well-being. Items in this self-report scale are rated on a five-point Likert-type scale response format from ‘*None of the time*’ (score of 1) to ‘*All of the time*’ (score of 5). All the responded items are added together and transformed into metric scores [28] to get the total response score, with higher scores indicating greater levels of mental well-being. The SWEMWBS has been validated in different languages including English [28,37], Swedish [33], Norwegian [33], and Danish [38] with acceptable psychometric properties. In the current study, the Swedish version of the SWEMWBS was used.

*General health*: adolescents' subjective assessment of their overall physical and mental well-being was measured using a single ad hoc item; “*How are you, in general*?” with response alternatives of *very bad, bad, neither good nor bad, good,* and *very good.* This item has a broad scope and serves as the translated version of the Swedish question “Hur mår du rent allmänt?". While it may provide insight into an individual's overall well-being, it may not be a validated measure specifically designed to assess general health. This single item has been employed with Swedish adolescents in prior research [46].

*COVID-19 pandemic impact*: The impact of COVID-19 was measured using eight ad hoc items. The items were “*How has the COVID-19 pandemic affected you in terms of ?”*: (i.e., studies, friends, parents, siblings, relatives, leisure, physical health, and mental health). All items were rated on a Likert-type scale ranging from 1 (much worse than before) to 5 (much better than before). Although the items were not intended to be used as a scale, the internal consistency was acceptable (α = 0.849).

### 2.3 Data analysis

The participants' characteristics (e.g., school year, gender, country of birth) were analyzed using descriptive statistics. The factor structure of the SWEMWBS was analyzed using Confirmatory Factor Analysis (CFA) and Rasch model analysis. The fit of the CFA model was evaluated using the Comparative Fit Index (CFI), Tucker Lewis Index (TLI), Root Mean Square Error of Approximation (RMSEA), and Standardized Root Mean Squared Residual (SRMR), with a good fitting model having CFI and TLI >0.95 with RMSEA and SRMR <0.06, including a non-significant χ2 test [47,48]. Due to the ordinal nature of the data, the CFAs were analyzed using the diagonally weighted least square estimator. The SWEMWBS was also examined in terms of internal consistency (i.e., Cronbach's α, and McDonald's ω), composite reliability, corrected item-total correlation, and average variance extracted (AVE). Cronbach's α and McDonald's ω > 0.7; composite reliability >0.6; corrected item-total correlation >0.4; and AVE >0.5 were acceptable to satisfactory [49].

The Rasch model analysis used information-weighted fit statistic (infit) mean square (MnSq) and outlier-sensitive fit statistics (outfit) MnSq to evaluate the fit for each item. Moreover, the fit between items and respondents to the unidimensional concept of the SWEMWBS was evaluated using item separation reliability, person separation reliability, the item separation index, and the person separation index. For the response scale of the SWEMWBS, the average and step measures of the difficulty in each point of the five-point Likert scale response were examined. The average and step measures are expected to monotonically increase to signify an acceptable response ordering [50]. Also, differential item functioning (DIF) of the Rasch analysis was used to examine the measurement invariance across gender and country of birth [30]. The Infit MnSq and Outfit MnSq values should be between 0.5 and 1.5 to indicate item fit [51]. The item separation reliability and person separation reliability should be larger than 0.7, and the item separation index and person separation index larger than 2 to indicate good item and respondent properties [52]. A DIF contrast of <0.5 signifies that there is no substantial DIF across the subgroupings [50].

Four nested models in the multigroupCFA (MGCFA) namely (i) a configural model, (ii) a model with factor loadings constrained equal, (iii) a model with factor loadings and item intercepts constrained equal, and (iv) a model with factor loadings, item intercepts and residuals constrained equal were constructed and compared to determine whether measurement invariance was supported [51,52]. Hence, measurement invariance is supported if a comparison between the more restrictive model and the less restrictive model shows the following indices: ΔCFI >0.01, ΔRMSEA <0.02, and ΔSRMR <0.03 for invariant loadings or <0.01 for invariant thresholds [48,53].

A network analysis was conducted to examine the relationships between different items of the SWEMWBS, COVID-19 impact and single-item self-reported general health. It comprised node and edge, where a node represented a variable (i.e., an item on the SWEMWBS, the items of the COVID-19 impact, and the single-item question on self-reported general health) and an edge represented a relationship between two variables. To conduct the network analysis, the Extended Bayesian Information Criterion Graphical Least Absolute Shrinkage and Selection Operator (EBICglasso estimator) was used [54]. Betweenness, Closeness, and Strength were computed to describe the centrality of nodes in the network. Betweenness refers to a measure of the number of times a node acts as a bridge between other nodes in the network, while Closeness measures how close a node is to all other nodes in the network. Strength represents a measure of the number of neighbors a node has and the number of connections (or ties) between them [54]]. Network analysis is considered as an alternative psychometric approach for assessing not only the structure of SWEMWBS but also for exploring the associations among its items. This feature allows us to understand how feelings and thoughts intercorrelated. Therefore, it helps not only for examining the potential mechanisms of psychological wellbeing but also for identification of core feelings/thoughts and understanding how they influence other feeling/thoughts [55]. Network analysis showed several advantages over simple Pearson correlation calculations among the SWEMWBS, COVID-19 impact, and a single-item self-reported general health. Unlike Pearson correlation, network analysis can handle nonlinear relationships between variables and detect indirect associations when working on complex multivariate relationships.

All the descriptive analyses and CFAs were conducted using the IBM SPSS 23.0 (IBM Corp., Armonk, NY) and the lavaan package in R software respectively [56] while the Rasch model analyses were conducted using WINSTEPS 3.75.0. Network analysis was conducted using JASP version 0.16.4.0.

## 3 Results

### 3.1 Scale-level psychometric properties of the SWEMWBS

The results from both CFA and Rasch analyses revealed acceptable psychometric properties of the SWEMWBS among all the participants. The internal consistency was also good (α = 0.900 and ω = 0.901). The CFA results revealed satisfactory model fit in the proposed fit statistics: CFI = 0.998, TLI = 0.997, RMSEA = 0.027, and SRMR = 0.027. Both AVE and Composite Reliability (CR) were above the recommended cut-off values. The psychometric properties obtained using Rasch analyses were satisfactory. Specifically, the item separation reliability was 1.0, the item separation index was 18.53, the person separation reliability was 0.83, and the person separation index was 2.19 (Table 2).

The eigenvalue for the total raw variance in observations was 16.102 (100%). The raw variances explained by measures, persons, and items were 9.102 (56.5%), 6.224 (38.7%), and 2.878 (17.9%) respectively. Also, the total raw unexplained variance was 7.0 (43.5%). The unexplained variance decreased gradually from the first contrast of 1.61 (10.0%) to the fifth contrast of 0.97 (6.0%). Additionally, the residual correlation between the items showed values less than 0.2, demonstrating local independence.

Findings at the item level of the SWEMWBS revealed that the CFA's factor loadings were statistically significant with standardized values ranging between 0.59 and 0.78. The item-total correlations were between 0.64 and 0.78. Similarly, Rasch analyses showed that all Infit MnSq values (ranging between 0.75 and 1.26) and Outfit MnSq values (ranging between 0.75 and 1.24) were acceptable, with the item difficulties ranging between −0.89 (item 7: *I've been able to make up my own mind about things*) and 0.64 (item 3: *I've been feeling relaxed*) (Table 1).

**Table 1:** Psychometric properties of the Short Warwick-Edinburgh Mental Well-Being Scale (SWEMWBS) in item level.

Item #	Analyses from Classical Test Theory				Rasch Analyses						
	Factor loading *^†^	**Item–total correlation**	**S**	**K**	**Infit MnSq**	**Outfit MnSq**	Difficulty	Discrimination	DIF contrast across gender^a^^¶^	DIF contrast across country of birth^b^	DIF contrast across school year^d^
SWEMWBS1	0.716	0.704	−0.903	0.544	1.02	1.03	−0.05	0.99	−0.16	−0.04	0
SWEMWBS2	0.771	0.756	−0.655	0.036	0.82	0.84	0.27	1.17	−0.08	−0.08	0.08
SWEMWBS3	0.757	0.686	−0.544	−0.348	1.11	1.12	0.64	0.86	0.44	−0.19	−0.07
SWEMWBS4	0.766	0.745	−0.743	0.247	0.86	0.86	0.25	1.15	0	−0.06	0
SWEMWBS5	0.780	0.778	−0.755	0.258	0.75	0.75	0.05	1.26	0	0	0
SWEMWBS6	0.677	0.658	−0.931	0.397	1.26	1.24	−0.26	0.77	−0.29	0.53	−0.08
SWEMWBS7	0.586	0.640	−1.154	1.241	1.20	1.15	−0.89	0.84	0.10	−0.12	0.12

a Gender: girls - boys.

b Country of birth: Swedish born vs non-Swedish born.

d School year: Year 9 in compulsory school vs Second grade of upper secondary school.

**Table 2:** Psychometric properties of the Short Warwick-Edinburgh Mental Well-Being Scale (SWEMWBS) in scale level.

Psychometric testing	Value
Cronbach's α Internal consistency	0.900
McDonald's omega	0.901
Confirmatory factor analysis	
χ^2^ (*df*)	66.218 (14)*
Comparative fit index	0.998
Tucker-Lewis index	0.997
Root-mean square error of approximation (90% CI)	0.027 (0.020–0.033)
Standardized root mean square residual	0.027
Average Variance Extracted	0.526
Composite Reliability	0.885
Item separation reliability from Rasch	1.0
Item separation index from Rasch	18.53
Person separation reliability from Rasch	0.83
Person separation index from Rasch	2.19
Variance explained by the Rasch factor	56.5%
Eigenvalue of the first residual (contrast) factor	0.974

*p < 0.001.

The five-point Likert scale in the SWEMWBS showed a monotonic increase in difficulty, with average measures ranging from −1.60 to 3.52, step measures ranging from −2.61 to 3.33, Infit MnSq values ranging from 0.90 to 1.28, and Outfit MnSq values ranging from 0.91 to 1.44 (Supplementary Table 1).

### 3.2 Psychometric properties of the SWEMWBS in DIF and measurement invariance

There was little to no DIF for the SWEMWBS items between genders, with values ranging from −0.29 to 0.44. Similarly, no substantial DIF was found between country of birth, with values ranging from −0.19 to 0, except for item 6 with a value of 0.53 (Table 1). Moreover, no substantial DIF was found for school year.

The findings from the measurement invariance analysis revealed that there were no differences between gender, country of birth, and school year as all the testing models across these groups were within acceptable parameters; CFI and TLI values were all higher than 0.95, and RMSEA and SRMR values were all less than 0.08. Moreover, the ΔCFI, ΔRMSEA, and ΔSRMR between every two nested models (i.e., M1 with M2, M2 with M3, and M3 with M4) were all within acceptable range. Therefore, measurement invariance across gender, country of birth, and school year was fully supported in terms of factor loadings, item intercepts, and residuals (Table 3).

**Table 3:** Measurement invariance of the Short Warwick-Edinburgh Mental Well-Being Scale (SWEMWBS) through confirmatory factor analysis.

Model and comparisons	Fit statistics							
	χ^2^ (df)	Δχ^2^ (Δdf)	CFI	ΔCFI	SRMR	ΔSRMR	RMSEA	ΔRMSEA
**Gender**								
M1: Configural	75.767 (28)		0.998		0.030		0.026 (0.019–0.033)	
M2: Metric	90.570 (34)		0.997		0.033		0.025 (0.019–0.032)	
M3: Scalar	184.519 (40)		0.993		0.037		0.037 (0.032–0.043)	
M4: Strict	236.786 (47)		0.990		0.048		0.039 (0.035–0.044)	
M2−M1	14.803 (6)			−0.001		0.003		−0.001
M3−M2	93.949 (6)			−0.004		0.004		0.012
M4−M3	52.267 (7)			−0.003		0.011		0.002
**Country of birth**								
M1: Configural	70.239(28)		0.998		0.028		0.024 (0.017–0.031)	
M2: metric	83.164 (34)		0.998		0.030		0.023 (0.017–0.030)	
M3: Scalar	108.366 (40)		0.997		0.028		0.025 (0.020–0.031)	
M4: Strict	134.206 (47)		0.996		0.031		0.026 (0.021–0.032)	
M2−M1	12.925 (6)			0		0.002		−0.001
M3−M2	25.202 (6)			−0.001		−0.002		0.002
M4−M3	25.84 (7)			−0.001		0.003		0.001
**Across school year**								
M1: Configural	71.722 (28)		0.998		0.028		0.024 (0.017–0.031)	
M2: metric	79.182 (34)		0.998		0.029		0.022 (0.016–0.029)	
M3: Scalar	83.548 (40)		0.998		0.026		0.020 (0.014–0.026)	
M4: Strict	89.702 (47)		0.998		0.028		0.019 (0.013–0.024)	
M2−M1	7.46 (6)			0		0.001		−0.002
M3−M2	4.366 (6)			0		−0.003		−0.002
M4−M3	6.154 (7)			0		0.002		−0.001

M1 = Model 1, a configural model; M2 = Model 2, a model based on M1 with all factor loadings constrained being equal across groups; M3 = Model 3, a model based on M2 with all item intercepts constrained being equal across groups; M4 = Model 4, a model based on M3 with all item residuals constrained being equal across groups.

CFI = comparative fit index; SRMR = standardized root mean square residual; RMSEA = root mean square error of approximation.

### 3.3 Network analysis of the SWEMWBS

**Fig. 1:**
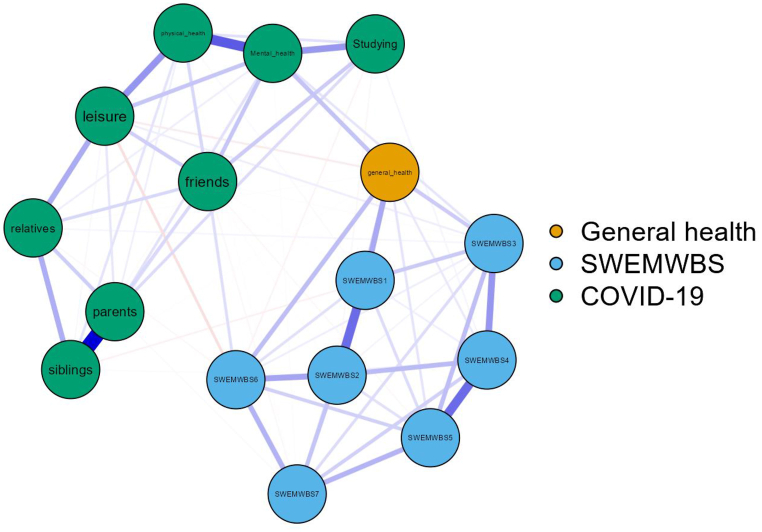
Network analysis of the Short Warwick-Edinburgh Mental Well-Being Scale (SWEMWBS) according to the relationships between mental health well-being and self-perceived general health among 5548 participants. **Note:** SWEMWB1 to 7 are the seven items of the Short Warwick-Edinburgh Mental Well-Being Scale.

The results showed 16 nodes in the network with 81 non-zero edges and a sparsity value of 0.33. The highest correlations between the SWEMWBS items were observed between Item 1 and Item 2 (r = 0.355), followed by Item 4 and Item 5 (r = 0.354). Additionally, the strongest correlations with general health were observed for Item 1 (r = 0.205) (Fig. 1). As Fig. 1 shows, general health can partly act as a bridge in the relationship between SWEMWBS items and COVID-19 impact. Self-reported general health was correlated with mental health (r = 0.144) but not with physical health. On the other hand, mental health was correlated with physical health (r = 0.395).

## 4 Discussion

The study aimed to examine the psychometric properties of the SWEMWBS among Swedish adolescents during the COVID-19 pandemic. The focus was on the measurement invariance of the SWEMWBS across gender and country of birth and its association with general health. The psychometric properties of the SWEMWBS scale among Swedish adolescents were found to be robust, demonstrating a unidimensional factor structure. This indicates that the scale measures a single construct of mental well-being, and scores can be aggregated for an overall assessment, with higher scores reflecting higher levels of mental health.

Moreover, the SWEMWBS showed robust psychometric properties including acceptable internal consistency and construct validity, which supports the findings reported by previous studies [20,30,33,37,38]. Furthermore, the item and person reliability suggested, respectively, good reproducibility of hierarchical item difficulty and person ordering on respondents’ ability. The item and person separation indices also suggested that the SWEMWBS is capable of separating items and persons into more than two distinct groups. These findings are consistent with the findings of previous studies [28,30] suggesting that the SWEMWBS also has robust psychometric properties among Swedish adolescents. Hence, school healthcare professionals and researchers alike can use the SWEMWBS to measure mental well-being at school levels.

The results of the measurement invariance analysis indicated that there were no differences between data for gender and country of birth as all the testing models across these groups were within an acceptable range, suggesting that these groups did not affect the interpretations of SWEMWBS items. Rasch analyses further supported measurement invariance of the SWEMWBS across gender and country of birth. That is, people with different genders or countries of birth had the same interpretation of the SWEMWBS items. However, Item 6 (i.e., *I've been feeling close to other people*) of the SWEMWBS was, according to country of birth, slightly more difficult for non-Swedish-born adolescents compared to Swedish-born individuals.

Research covering both the international scene as well as Sweden suggests that ethnic minority groups are more likely to experience health problems [57,58]. Immigrant adolescents and families commonly face challenges such as discrimination, cultural conflicts, low self-esteem, language difficulties, and strained parent-child relationships, leading to feelings of loneliness [59,60]. Research supports this by showing a strong correlation between migration status and mental health issues among adolescents [61]. Despite the difficulties posed by item 6 on the SWEMWBS for non-Swedish-born adolescents, it is recommended to keep the item due to its psychometric support from CFA results. Taken the results together, it can be concluded that there is no significant invariance between subgroups on the SWEMWBS and so clinicians and researchers can confidently use the SWEMWBS to compare mental well-being between Swedish adolescents who belong to different subgroups (i.e., gender: males vs. females, and country of birth: Swedish-born vs. non-Swedish-born).

The network analysis results showed positive, significant correlations between SWEMWBS items and general health. These associations are supported by other studies [34,62]. The findings have significance, emphasizing the need for increased focus on improving mental well-being among Swedish adolescents. Regardless of whether there is an ongoing pandemic or not, the need for increased focus on improving mental health and well-being among adolescents is clear. Over the past 30 years, the proportion of adolescents, both girls and boys, aged 15, with mental health problems (such as headaches, feeling down, irritable, with bad moods, feeling nervous, having difficulty sleeping) has increased. Sweden is above average in mental illness among European and North American countries, while the neighboring Nordic countries are below average [63]. The development of adolescents' mental health and well-being is important to follow up and promote, both for clinicians, such as health professionals in school healthcare as well as youth clinics, public health workers and researchers. The SWEMWBS can provide a basis for the salutogenic perspective as it is important to strengthen the positive health aspects for adolescents’ overall health and functioning [64]. Further research is needed to evaluate the feasibility of the SWEMWBS in clinical practice.

### 4.1 Limitations

This study presents potential limitations as a cross-sectional survey design was adopted. Limitations pertaining to this include the fact that establishing cause-and-effect relationships is not possible. Hence, readers should take caution in overly extending the findings of this study. Closely related to the design of the study are the instruments used for data collection. Self-report measures were used in this study and so the responses obtained may be prone to some biases such as social desirability response bias. Moreover, the study primarily focused on factorial validity/internal structure and internal consistency reliability; other relevant psychometric properties such as concurrent validity, discriminant validity, convergent validity, predictive validity, known-group validity, and test-retest reliability were not investigated. The method of data collection is also another limitation of the study. The data was gathered via an online survey. Therefore, researchers had little control over the research environment. Social desirability is another potential limitation of the study. Given that self-reporting on the SWEMWBS can be a sensitive matter, adolescents may want to overestimate positive mental well-being. The results of the study may have limited generalizability to the entire Swedish adolescents because the data was collected from Jönköping County. Test-retest reliability was not measured in this study. Therefore, future studies should consider measuring the stability of the SWEMWBS over time. Finally, due to the lack of clinical data, the present study did not calculate sensitivity and specificity for the SWEMWBS although the study identified people who may be at risk of mental health problems.

Although this study was conducted in the context of a pandemic, its importance and usefulness extend beyond COVID-19 challenges. SWEMWBS, as an instrument measuring wellbeing, could be used more generally in a variety of human service organizations, such as youth clinics, schools, and social services in the sense that it forms a basis for a dialogue between professionals and adolescents. Also, since the SWEMWBS scale has a salutogenic perspective, it can work as an essential tool in promoting positive mental health and well-being in relation to adolescents' functioning. In addition, SWEMWBS could also be used in health screening and research, where also its clinical implication can be further studied.

## 5 Conclusions

This study demonstrated that the SWEMWBS had acceptable psychometric properties as well as a unidimensional structure capable of assessing mental well-being among Swedish adolescents. Furthermore, the SWEMWBS was invariant across gender and country of birth, which suggests that data across these subgroups can comfortably be compared. Additionally, the SWEMWBS was positively correlated with general health, which further suggests that there should be conscious efforts by school healthcare professionals and other regulating authorities to promote health and well-being.

## Funding

There is no funding associated with this research.

## Data availability statement

Data will be made available on reasonable request.

## CRediT authorship contribution statement

**Amir H. Pakpour:** Writing – review & editing, Writing – original draft, Validation, Methodology, Investigation, Formal analysis, Conceptualization. **Marit Eriksson:** Writing – review & editing, Supervision, Software, Resources, Project administration, Methodology, Investigation, Formal analysis. **Ida Erixon:** Writing – review & editing, Software, Resources, Project administration, Methodology, Investigation. **Anders Broström:** Writing – review & editing, Validation, Supervision, Methodology, Conceptualization. **Staffan Bengtsson:** Writing – review & editing, Validation, Supervision, Methodology, Conceptualization. **Malin Jakobsson:** Writing – review & editing, Validation, Supervision, Software. **Karina Huus:** Writing – review & editing, Validation, Supervision, Software, Methodology, Investigation, Conceptualization.

## Declaration of competing interest

The authors declare that they have no known competing financial interests or personal relationships that could have appeared to influence the work reported in this paper.
