# Supplementary material for: The Short Warwick-Edinburgh Mental Well-being Scale (SWEMWBS) - A psychometric evaluation of adolescents in Sweden during the COVID-19 pandemic
Source: Heliyon. 2024 Mar 5;10(6):e27620. doi: 10.1016/j.heliyon.2024.e27620 (PMC10950601; doi:10.1016/j.heliyon.2024.e27620)
Supplement: Multimedia component 1 [file mmc1.docx]

Supplementary Tables

**Supplementary Table 1.** Response disordering tests of the Short Warwick-Edinburgh Mental Well-Being Scale (SWEMWBS).

| SWEMWBS | Average measure | Step measure | Infit MnSq | Outfit MnSq |
| --- | --- | --- | --- | --- |
| 1 | -1.60 | - | 1.28 | 1.44 |
| 2 | -0.67 | -2.61 | 1.02 | 1.08 |
| 3 | 0.43 | -1.14 | 0.91 | 0.91 |
| 4 | 1.88 | 0.42 | 0.90 | 0.91 |
| 5 | 3.52 | 3.33 | 1.08 | 1.03 |


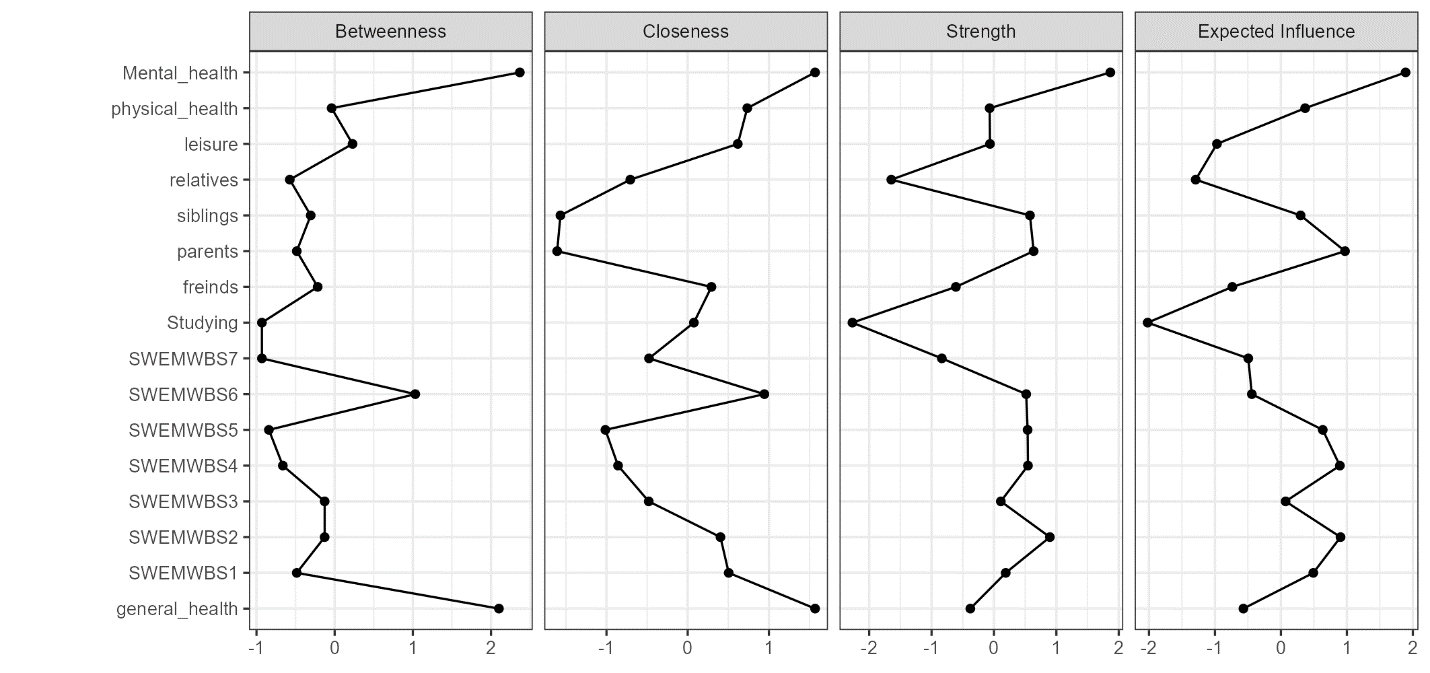


**Supplementary Figure 1.** Centrality Plots for EBICglaaso network depicting the betweenness, closeness, and degree (strength) of each node (variable) among 5548 Swedish adolescents. Note: SWEMWB1 to 7 are seven items of the Short Warwick-Edinburgh Mental Well-Being Scale.

| **Supplementary Table 2. Centrality measures per variable** | | | | | | | | | |
| --- | --- | --- | --- | --- | --- | --- | --- | --- | --- |
|  | | **Network** | | | | | | | |
| **Variable** | | **Betweenness** | | **Closeness** | | **Strength** | | **Expected influence** | |
| general_health |  | 2.101 |  | 1.569 |  | -0.379 |  | -0.569 |  |
| SWEMWBS1 |  | -0.485 |  | 0.505 |  | 0.189 |  | 0.491 |  |
| SWEMWBS2 |  | -0.128 |  | 0.403 |  | 0.896 |  | 0.904 |  |
| SWEMWBS3 |  | -0.128 |  | -0.480 |  | 0.110 |  | 0.071 |  |
| SWEMWBS4 |  | -0.663 |  | -0.858 |  | 0.545 |  | 0.894 |  |
| SWEMWBS5 |  | -0.842 |  | -1.014 |  | 0.539 |  | 0.634 |  |
| SWEMWBS6 |  | 1.031 |  | 0.944 |  | 0.517 |  | -0.441 |  |
| SWEMWBS7 |  | -0.931 |  | -0.476 |  | -0.836 |  | -0.495 |  |
| Studying |  | -0.931 |  | 0.076 |  | -2.269 |  | -2.020 |  |
| freinds |  | -0.217 |  | 0.293 |  | -0.610 |  | -0.737 |  |
| parents |  | -0.485 |  | -1.607 |  | 0.636 |  | 0.972 |  |
| siblings |  | -0.307 |  | -1.567 |  | 0.577 |  | 0.299 |  |
| relatives |  | -0.574 |  | -0.707 |  | -1.646 |  | -1.292 |  |
| leisure |  | 0.229 |  | 0.617 |  | -0.064 |  | -0.970 |  |
| physical_health |  | -0.039 |  | 0.734 |  | -0.069 |  | 0.367 |  |
| Mental_health |  | 2.369 |  | 1.569 |  | 1.865 |  | 1.890 |  |
|  | | | | | | | | | |

| **Supplementary Table 3. Weights matrix among items** | | | | | | | | | | | | | | | | | | | | | | | | | | | | | | | | | |
| --- | --- | --- | --- | --- | --- | --- | --- | --- | --- | --- | --- | --- | --- | --- | --- | --- | --- | --- | --- | --- | --- | --- | --- | --- | --- | --- | --- | --- | --- | --- | --- | --- | --- |
|  | | **Network** | | | | | | | | | | | | | | | | | | | | | | | | | | | | | | | |
| **Variable** | | **general_health** | | **SWEMWBS1** | | **SWEMWBS2** | | **SWEMWBS3** | | **SWEMWBS4** | | **SWEMWBS5** | | **SWEMWBS6** | | **SWEMWBS7** | | **Studying** | | **freinds** | | **parents** | | **siblings** | | **relatives** | | **leisure** | | **physical_health** | | **Mental_health** | |
| general_health |  | 0.000 |  | 0.205 |  | 0.090 |  | 0.125 |  | 0.053 |  | 0.064 |  | 0.156 |  | 0.000 |  | 0.000 |  | 0.000 |  | 0.000 |  | -0.007 |  | -0.009 |  | -0.045 |  | 0.003 |  | 0.144 |  |
| SWEMWBS1 |  | 0.205 |  | 0.000 |  | 0.355 |  | 0.127 |  | 0.036 |  | 0.085 |  | 0.075 |  | 0.085 |  | 0.000 |  | -0.006 |  | 0.000 |  | 0.000 |  | 0.000 |  | -0.003 |  | 0.014 |  | 0.000 |  |
| SWEMWBS2 |  | 0.090 |  | 0.355 |  | 0.000 |  | 0.026 |  | 0.164 |  | 0.073 |  | 0.217 |  | 0.140 |  | -0.020 |  | -0.007 |  | 0.000 |  | 0.011 |  | 0.000 |  | 0.000 |  | 0.000 |  | 0.000 |  |
| SWEMWBS3 |  | 0.125 |  | 0.127 |  | 0.026 |  | 0.000 |  | 0.251 |  | 0.155 |  | 0.041 |  | 0.085 |  | 0.000 |  | -0.009 |  | 0.000 |  | -0.033 |  | 0.023 |  | 0.041 |  | 0.000 |  | 0.064 |  |
| SWEMWBS4 |  | 0.053 |  | 0.036 |  | 0.164 |  | 0.251 |  | 0.000 |  | 0.354 |  | 0.049 |  | 0.115 |  | 0.024 |  | 0.000 |  | 0.000 |  | 0.000 |  | 0.000 |  | 0.000 |  | 0.000 |  | 0.000 |  |
| SWEMWBS5 |  | 0.064 |  | 0.085 |  | 0.073 |  | 0.155 |  | 0.354 |  | 0.000 |  | 0.113 |  | 0.179 |  | 0.000 |  | -0.019 |  | 0.000 |  | -0.005 |  | 0.000 |  | 0.000 |  | 0.000 |  | 0.000 |  |
| SWEMWBS6 |  | 0.156 |  | 0.075 |  | 0.217 |  | 0.041 |  | 0.049 |  | 0.113 |  | 0.000 |  | 0.183 |  | -0.026 |  | 0.080 |  | 0.000 |  | 0.000 |  | -0.019 |  | -0.075 |  | 0.009 |  | 0.000 |  |
| SWEMWBS7 |  | 0.000 |  | 0.085 |  | 0.140 |  | 0.085 |  | 0.115 |  | 0.179 |  | 0.183 |  | 0.000 |  | 0.000 |  | -0.019 |  | 0.012 |  | 0.000 |  | 0.000 |  | 0.000 |  | 0.004 |  | 0.008 |  |
| Studying |  | 0.000 |  | 0.000 |  | -0.020 |  | 0.000 |  | 0.024 |  | 0.000 |  | -0.026 |  | 0.000 |  | 0.000 |  | 0.125 |  | 0.086 |  | 0.001 |  | 0.000 |  | 0.001 |  | 0.074 |  | 0.247 |  |
| freinds |  | 0.000 |  | -0.006 |  | -0.007 |  | -0.009 |  | 0.000 |  | -0.019 |  | 0.080 |  | -0.019 |  | 0.125 |  | 0.000 |  | 0.066 |  | 0.093 |  | 0.095 |  | 0.117 |  | 0.095 |  | 0.136 |  |
| parents |  | 0.000 |  | 0.000 |  | 0.000 |  | 0.000 |  | 0.000 |  | 0.000 |  | 0.000 |  | 0.012 |  | 0.086 |  | 0.066 |  | 0.000 |  | 0.610 |  | 0.120 |  | 0.059 |  | 0.035 |  | 0.073 |  |
| siblings |  | -0.007 |  | 0.000 |  | 0.011 |  | -0.033 |  | 0.000 |  | -0.005 |  | 0.000 |  | 0.000 |  | 0.001 |  | 0.093 |  | 0.610 |  | 0.000 |  | 0.195 |  | 0.018 |  | 0.066 |  | -0.012 |  |
| relatives |  | -0.009 |  | 0.000 |  | 0.000 |  | 0.023 |  | 0.000 |  | 0.000 |  | -0.019 |  | 0.000 |  | 0.000 |  | 0.095 |  | 0.120 |  | 0.195 |  | 0.000 |  | 0.202 |  | 0.000 |  | 0.040 |  |
| leisure |  | -0.045 |  | -0.003 |  | 0.000 |  | 0.041 |  | 0.000 |  | 0.000 |  | -0.075 |  | 0.000 |  | 0.001 |  | 0.117 |  | 0.059 |  | 0.018 |  | 0.202 |  | 0.000 |  | 0.254 |  | 0.136 |  |
| physical_health |  | 0.003 |  | 0.014 |  | 0.000 |  | 0.000 |  | 0.000 |  | 0.000 |  | 0.009 |  | 0.004 |  | 0.074 |  | 0.095 |  | 0.035 |  | 0.066 |  | 0.000 |  | 0.254 |  | 0.000 |  | 0.395 |  |
| Mental_health |  | 0.144 |  | 0.000 |  | 0.000 |  | 0.064 |  | 0.000 |  | 0.000 |  | 0.000 |  | 0.008 |  | 0.247 |  | 0.136 |  | 0.073 |  | -0.012 |  | 0.040 |  | 0.136 |  | 0.395 |  | 0.000 |  |
|  | | | | | | | | | | | | | | | | | | | | | | | | | | | | | | | | | |


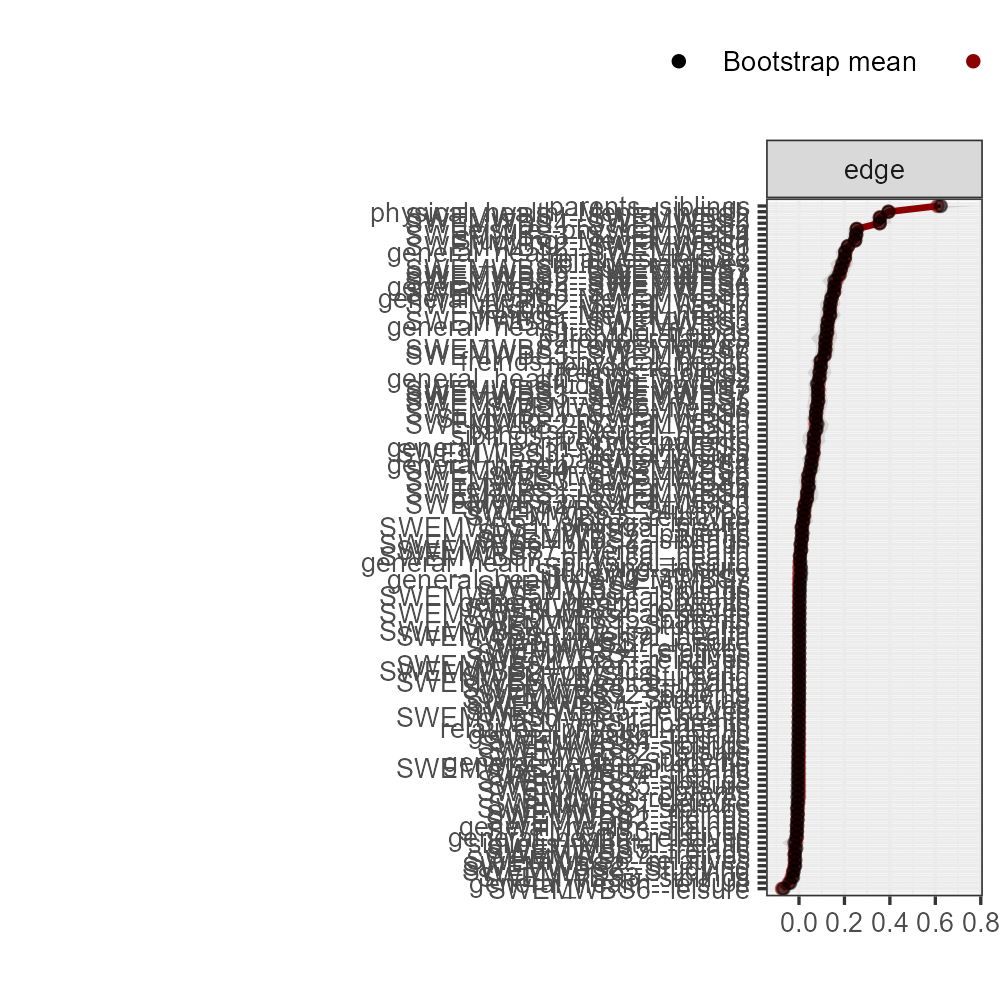


**Supplementary Figure 2**. Edge Stability Plot for Short Warwick-Edinburgh Mental Well-Being Scale (SWEMWBS) Network Analysis


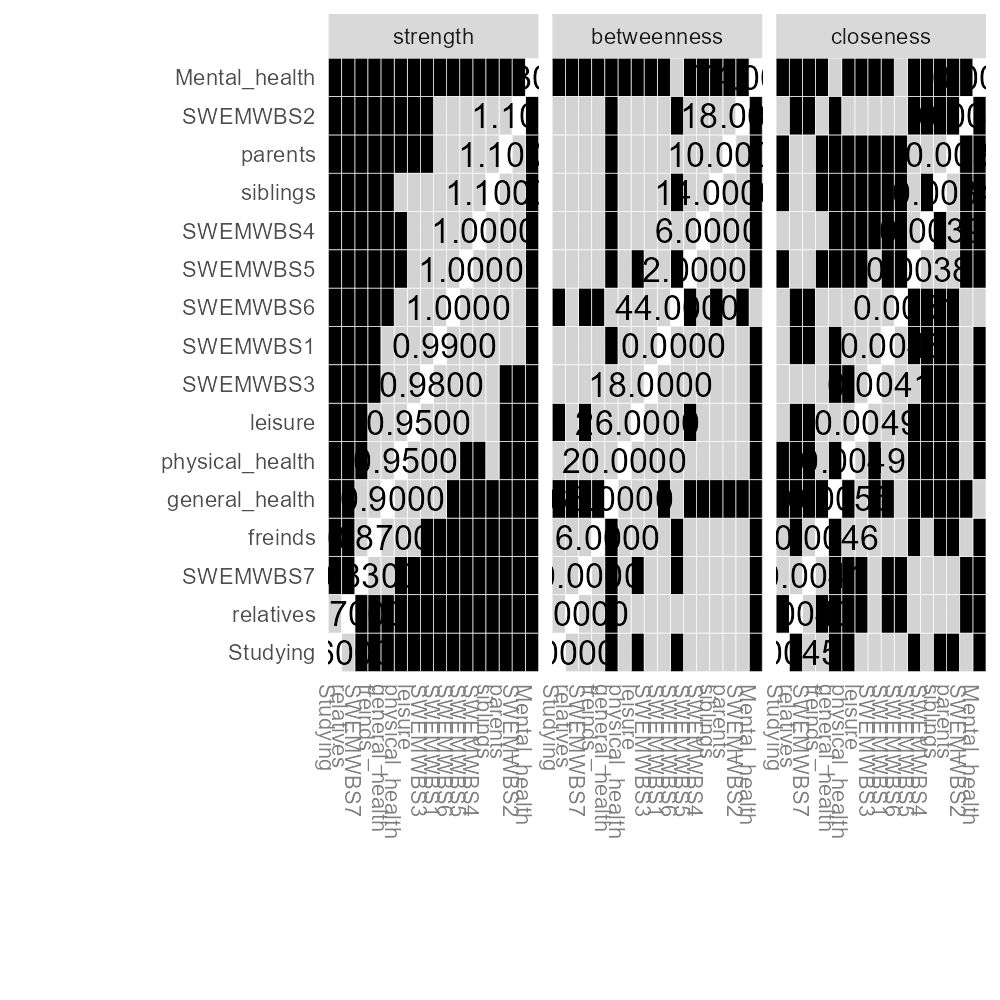


**Supplementary Figure 3**. Centrality Stability Plot for Network Analysis
