# Supplementary material for: The Short Warwick-Edinburgh Mental Well-being Scale (SWEMWBS) - A psychometric evaluation of adolescents in Sweden during the COVID-19 pandemic
Source: Heliyon. 2024 Mar 5;10(6):e27620. doi: 10.1016/j.heliyon.2024.e27620 (PMC10950601; doi:10.1016/j.heliyon.2024.e27620)
Supplement: Multimedia component 2 [file mmc2.docx]

**Section A: Sociodemographic Information**

1. Sex: Female [] Male [ ]
2. Country of birth:

**Section B: The Short Warwick–Edinburgh Mental Well-being Scale (SWEMWBS)**

Please respond by ticking (√) one of the responses provided that reflect your experience of each over the last 2 weeks.

*Response options:*

1=None of the time

2=Rarely

3=Some of the times

4=often

5=all of the time

|  | **None of the time** | **Rarely** | **Some of the times** | **Often** | **all of the time** |
| --- | --- | --- | --- | --- | --- |
| I’ve been feeling optimistic about the future |  |  |  |  |  |
| I’ve been feeling useful |  |  |  |  |  |
| I’ve been feeling relaxed |  |  |  |  |  |
| I’ve been dealing with problems well |  |  |  |  |  |
| I’ve been thinking clearly |  |  |  |  |  |
| I’ve been feeling close to other people |  |  |  |  |  |
| I’ve been able to make up my own mind about things |  |  |  |  |  |

**Section C: general health**

How are you in general?

*Response options:*

1= very bad 2= bad 3= neither good nor bad 4= good 5= very good

**Section D: COVID-19 pandemic impact**

Please respond by ticking (√) one of the responses provided your feelings and thoughts.

*Response options:*

1= much worse

2= worse

3= same (neither worse nor better)

4= better

5= much better

| *how has the COVID-19 pandemic affected you in terms of…* | **much worse** | **Worse** | **Same** | **Better** | **much better** |
| --- | --- | --- | --- | --- | --- |
| Studies |  |  |  |  |  |
| Friends |  |  |  |  |  |
| Parents |  |  |  |  |  |
| Siblings |  |  |  |  |  |
| Leisure |  |  |  |  |  |
| Physical health |  |  |  |  |  |
| Mental health |  |  |  |  |  |
